# Children's Country Holidays Fund

**Published:** 1916-06-24

**Authors:** 

**Affiliations:** Hon. Treasurer, C.C.H.F.


*To the Editor of* THE HOSPITAL.

### Children's Country Holidays Fund.

*To the Editor of* THE HOSPITAL.

SIR,—As Hon. Treasurer of the Children's Country Holidays Fund, I venture to ask the hospitality of your columns in order to make an appeal for assistance.

Ailing children from the elementary day schools of London are sent to the country for a fortnight during the summer holiday, and return with renewed health and vigour which they would otherwise never enjoy.

Subscriptions have fallen very considerably owing to the war, and although the many difficulties engendered by war-time conditions have made it impossible for the Fund to work on its usual scale, money is urgently needed to send away even the much reduced number of children. There is also a great dearth of country homes for these children, and the Secretary would be glad to hear of villages where they could be housed. All subscriptions and donations will be gratefully received and acknowledged here.—I am, Sir, your obedient servant,

ARRAN, Hon. Treasurer, C.C.H.F.

